# Protein disulfide isomerase does not act as an unfoldase in the disassembly of cholera toxin

**DOI:** 10.1042/BSR20181320

**Published:** 2018-09-07

**Authors:** Patrick Cherubin, Jessica Guyette, Michael Taylor, Morgan O’Donnell, Laura Herndon, Helen Burress, Aladdin Riad, Suren A. Tatulian, Ken Teter

**Affiliations:** 1 Burnett School of Biomedical Sciences, College of Medicine, University of Central Florida, Orlando, FL, U.S.A.; 2 Department of Physics, University of Central Florida, Orlando, FL, U.S.A.

**Keywords:** cholera toxin, molecular chaperones, protein disulfide isomerase

## Abstract

Cholera toxin (CT) is composed of a disulfide-linked A1/A2 heterodimer and a ring-like, cell-binding B homopentamer. The catalytic A1 subunit must dissociate from CTA2/CTB_5_ to manifest its cellular activity. Reduction of the A1/A2 disulfide bond is required for holotoxin disassembly, but reduced CTA1 does not spontaneously separate from CTA2/CTB_5_: protein disulfide isomerase (PDI) is responsible for displacing CTA1 from its non-covalent assembly in the CT holotoxin. Contact with PDI shifts CTA1 from a protease-resistant conformation to a protease-sensitive conformation, which is thought to represent the PDI-mediated unfolding of CTA1. Based solely on this finding, PDI is widely viewed as an ‘unfoldase’ that triggers toxin disassembly by unfolding the holotoxin-associated A1 subunit. In contrast with this unfoldase model of PDI function, we report the ability of PDI to render CTA1 protease-sensitive is unrelated to its role in toxin disassembly. Multiple conditions that promoted PDI-induced protease sensitivity in CTA1 did not support PDI-mediated disassembly of the CT holotoxin. Moreover, preventing the PDI-induced shift in CTA1 protease sensitivity did not affect PDI-mediated disassembly of the CT holotoxin. Denatured PDI could still convert CTA1 into a protease-sensitive state, and equal or excess molar fractions of PDI were required for both efficient conversion of CTA1 into a protease-sensitive state and efficient disassembly of the CT holotoxin. These observations indicate the ‘unfoldase’ property of PDI does not play a functional role in CT disassembly and does not represent an enzymatic activity.

## Introduction

Protein disulfide isomerase (PDI) is a dynamic, flexible molecule with an abb′xa′c domain organization that is arranged in a horseshoe configuration [1]. The 57-kDa protein contains two enzymatically active thioredoxin-like domains (a and a′), two inactive thioredoxin-like domains (b and b′), an x linker, and an acidic c domain [2,3]. It is mainly located in the endoplasmic reticulum (ER) but also functions at other intracelluar and extracellular locations [4,5]. As an oxidoreductase, PDI can assist proper protein folding through oxidation, reduction, and isomerase activities [6]. As a chaperone, PDI can prevent the aggregation of misfolded proteins [7,8]. The oxidoreductase and chaperone functions of PDI are complementary, but they are also distinct [9,10] and can be distinguished by drug treatment: the peptide antibiotic bacitracin only inhibits the reductase activity of PDI [11,12], while the aminoglycoside antibiotic ribostamycin specifically inhibits the chaperone activity of PDI by an unknown mechanism [13]. The redox status of PDI can also influence its function, as only the reduced form of PDI will interact with select substrates [14,15]. The oxidoreductase and chaperone functions of PDI allow it to play an essential role in ER quality control by facilitating the proper folding and assembly of nascent secretory proteins.

PDI is also involved with cholera intoxication. Cholera toxin (CT) is a prototypical AB_5_ protein toxin consisting of an enzymatically active A domain and a homopentameric cell-binding B domain [16]. The A subunit is proteolytically nicked to generate an A1/A2 heterodimer that remains covalently linked by a single disulfide bond near the C-terminus of CTA1 and the N-terminus of CTA2. The ADP-ribosyltransferase activity of the toxin is present in the 21-kDa A1 subunit, while the 5-kDa A2 subunit extends into the central pore of the ring-like B pentamer and thereby links the enzymatic and cell-binding components of the toxin (Supplementary Figure S1). The CT holotoxin binds to GM1 gangliosides on the host plasma membrane, triggering toxin endocytosis and retrograde transport to the ER [17,18]. Reduction of the CTA1/CTA2 disulfide bond in the ER permits the dissociation of CTA1 from CTA2/CTB_5_ [19,20]. The free CTA1 subunit then shifts to a disordered conformation that facilitates its ER-to-cytosol export through a membrane-spanning translocon pore [21]. However, reduced CTA1 does not spontaneously separate from CTA2/CTB_5_: extensive non-covalent contacts within the holotoxin maintain a stable association between CTA1 and the rest of the toxin [22,23]. The reduced form of PDI is instead responsible for dislodging CTA1 from its non-covalent assembly in the CT holotoxin. This event does not require the oxidoreductase activity of PDI and specifically involves the reduced form of PDI, as oxidized PDI does not bind to CTA1 [24–26]. Other ER-localized oxidoreductases (ERp57 and ERp72) cannot disassemble CT [24], and PDI-deficient cells are completely resistant to CT [26]. The molecular mechanism underlying PDI-mediated CT disassembly remains a matter of debate.

In 2001, contact with reduced PDI was shown to convert either free or holotoxin-associated CTA1 from a protease-resistant conformation into a protease-sensitive conformation [25]. As unfolded proteins are generally more sensitive to proteolysis than folded variants of the same protein, this conformational shift was interpreted as the PDI-mediated unfolding of CTA1. For holotoxin-associated CTA1, the shift to a protease-sensitive state was concomitant with its release from the rest of the toxin [25]. It, therefore, appeared that the unfolding of holotoxin-associated CTA1 was responsible for its displacement from CTA2/CTB_5_. Although no direct structural evidence was presented to document CTA1 unfolding by PDI and no mechanistic basis for this phenomenon was proposed, PDI has since been viewed as an ‘unfoldase’.

A series of biophysical studies have provided solid evidence supporting an alternative role for PDI in CT disassembly. CD, fluorescence spectroscopy, Fourier transform IR (FTIR) spectroscopy, and differential scanning calorimetry have collectively shown the free but not holotoxin-associated CTA1 subunit is an unstable protein with a disordered structure at the physiological temperature of 37°C [26–29]. Separation of CTA1 from the CT holotoxin would consequently allow CTA1 to unfold at physiological temperature. As such, PDI does not need to actively unfold CTA1 – the toxin will spontaneously shift to a disordered conformation upon its removal from CTA2/CTB_5_. In fact, the spontaneous unfolding of CTA1 at 37°C will displace its PDI binding partner [26]. Experiments using isotope-edited FTIR spectroscopy further demonstrated that PDI does not unfold CTA1, with unfolding defined as a substantial gain in disordered structure at the expense of α-helical and/or β-sheet structures [26]. Instead, PDI itself shifted to an unfolded conformation upon contact with either free or holotoxin-associated CTA1 [24]. The substrate-induced unfolding of PDI provides a molecular explanation for PDI-mediated holotoxin disassembly: binding of PDI to CT results in the partial unfolding of PDI, which promotes CTA1 dissociation by acting as a wedge between CTA1 and the rest of the toxin.

The ‘wedge’ model for CT disassembly has been supported by substantial biochemical and biophysical data. Holotoxin disassembly did not occur when PDI was locked in a folded conformation by treatment with the intramolecular cross-linker 1-Ethyl-3-(3-dimethylaminopropyl)carbodiimide (EDC) or when its substrate-induced unfolding was blocked by ribostamycin treatment [24]. Bacitracin-treated PDI could still unfold in the presence of CTA1 and could still separate CTA1 from the reduced holotoxin [24], which confirmed the reductase activity of PDI is not required for CT disassembly [25]. *In vivo* translocation events downstream of CT disassembly did not appear to require PDI: a CTA1 construct expressed directly in the ER of transfected cells was exported to the cytosol of PDI-deficient cells [26] and ribostamycin-treated cells [24]. Based on these collective results, we proposed an alternative model for CT disassembly in which the substrate-induced unfolding of PDI provides a mechanistic basis for the separation of CTA1 from CTA2/CTB_5_.

It is possible that a subtle, PDI-induced change in CTA1 tertiary structure (as detected by protease sensitivity) is more important for CT disassembly than the substrate-induced unfolding of PDI. To examine this issue, we used several experimental conditions to look at correlations between the PDI-induced shift in CTA1 protease sensitivity and the PDI-mediated release of CTA1 from CTA2/CTB_5_. No method other than the protease sensitivity assay has been used in experimental support of the unfoldase model; ‘unfoldase’ is synonymous with shifting CTA1 to a protease-sensitive state. We therefore focussed on the link between protease sensitivity and toxin disassembly. Using two different proteases, we found equimolar or excess PDI was required to fully convert CTA1 into a protease-sensitive state. Efficient disassembly of the CT holotoxin by PDI likewise required a molar excess of PDI over substrate. The inability to efficiently shift CTA1 to a protease-sensitive conformation and disassemble the CT holotoxin at sub-stoichiometric molar ratios of PDI:substrate suggested a non-enzymatic mechanism for the unfoldase function of PDI, which could explain why previous studies have used an approximately 50-fold or greater molar excess of PDI to study its toxin unfoldase activity [25,30–32]. The conversion of CTA1 into a protease-sensitive state by denatured PDI further supported a non-enzymatic mechanism for the unfoldase property of PDI. Moreover, we found no correlation between CT disassembly and the PDI-induced shift in CTA1 protease sensitivity (i.e. the putative unfolding of CTA1 by PDI): denatured PDI, ribostamycin-treated PDI, and EDC-treated PDI could each convert CTA1 into a protease-sensitive state but could not displace reduced CTA1 from its holotoxin. We also noted that 10% glycerol blocked the PDI-induced conversion of CTA1 into a protease-sensitive state but did not inhibit CT disassembly. Thus, the proposed unfoldase activity of PDI does not represent an enzymatic property of PDI and is not functionally linked to CT disassembly.

## Materials and methods

### Protease sensitivity assay

CT or the disulfide-linked CTA1/CTA2 heterodimer (Sigma–Aldrich, St. Louis, MO) was reduced in 0.02 M NaPO_4_ buffer (pH 7.4) with 1 mM GSH. Toxin samples (200 ng CT or 1 μg CTA1/CTA2) were aliquoted in 20 μl and incubated at 25, 30, or 37°C for 1 h in the presence of various concentrations of PDI (Sigma–Aldrich). When indicated, 10% glycerol or 0.1 mM ribostamycin (Sigma–Aldrich) was also present in the assay buffer. All samples were then placed on ice for 10 min, followed by 1 h at 4°C with 0.04 mg/ml of thermolysin or 0.1 mg/ml of trypsin. The stock of trypsin was treated with N-tosyl-l-phenylalanyl chloromethyl ketone by the manufacturer (Sigma–Aldrich) to inhibit the activity of potentially contaminating chymotrypsin. After adding 5 μl of 4× sample buffer to each reaction, the proteins were resolved using SDS/PAGE with 15% polyacrylamide gels and visualized by Coomassie stain or Western blot. This general protocol replicates the procedure used in several previous publications [25,30,31] in order to allow direct comparison between those reports and the current study. Oxidized PDI could not be used for these studies because it does not bind to CTA1 [25,26]. Disulfide-linked CTA1/CTA2 could not be used for these studies because the covalent association of CTA1 with CTA2 retains CTA1 in a folded, protease-resistant conformation even at 37°C [27]. In some experiments, PDI was pre-heated at 90°C for 5 min or pre-treated with 200 mM EDC (Fisher Scientific, Waltham, MA) for 30 min at 25°C. EDC was removed from the PDI sample by dialysis with a 3500 MWCO membrane before using the EDC-treated PDI, and the absence of cross-linked PDI oligomers or aggregates was confirmed by SDS/PAGE.

### Western blot

For trypsin-based protease sensitivity assays, CTA1 was detected by Western blot. A primary rabbit polyclonal anti-CT antibody (Sigma–Aldrich) was used at a 1:5000 dilution for a 4°C overnight incubation. After four 5-min washes with 1% milk in TBS containing 0.1% Tween-20 (TBS-T), the membrane was incubated in a 1:10000 dilution of Peroxidase AffiniPure goat anti-rabbit IgG antibody (Jackson ImmunoResearch, West Grove, PA) for 30 min at room temperature. After two 5-min washes with 1% milk in TBS-T and two 5-min washes with TBS-T, the membrane was soaked in ECL reagent and exposed to film.

### Holotoxin disassembly monitored by surface plasmon resonance (SPR)

As previously described [33], the CT holotoxin (Sigma–Aldrich) was appended to an SPR sensor slide coated with ganglioside GM1 (Calbiochem, La Jolla, CA). PBS (pH 7.4) with 0.05% Tween 20 (PBS-T) was perfused over the CT sensor for 10 min at 30°C with a 41 μl/min flow rate to establish a baseline refractive index unit (RIU) signal corresponding to the mass of the bound holotoxin. A PBS-T solution containing 1 mM GSH and PDI, ribostamycin-treated PDI, EDC-treated PDI, or heat-denatured PDI was then perfused over the CT sensor at 30°C and a flow rate of 41 μl/min. When indicated, 10% glycerol was also present in the perfusion buffer. The CTA1/CTA2 disulfide bond in the CT holotoxin is reduced by 1 mM GSH (Supplementary Figure S2), so the oxidoreductase activity of PDI (which may have been disrupted by our treatments) was not required for holotoxin reduction in these assays. As indicated, PDI was heated to 90°C for 5 min, pre-treated with 200 mM EDC for 30 min at 25°C, or incubated with 0.1 mM ribostamycin. PDI samples were removed from the perfusion buffer after approximately 300 s and replaced with sequential additions of anti-PDI (Enzo Life Sciences, Farmingdale, NY), monoclonal anti-CTA1 [34], and anti-CTB (Sigma–Aldrich) antibodies at 1:10000, 1:500, and 1:15000 dilutions, respectively. Experiments were performed with a Reichert (Depew, NY) dual-channel SR7000 SPR refractometer. CT holotoxin was present in both channels, but only one channel was exposed to experimental conditions. The untreated, CT-containing channel was used to monitor potential baseline drift in the experimental samples.

### ELISA-based holotoxin disassembly assay

GM1 at a concentration of 3 μg/ml in 200 proof ethanol was added to each well of a 96-well ELISA plate. The 50-μl volume was allowed to completely evaporate over the course of 90 min in a fume hood. The wells were then washed three times with TBS-T, blocked with 5% BSA in TBS for 1 h at room temperature, washed another three times with TBS-T, and exposed to 50 μl of CT at a concentration of 5 μg/ml. After 1 h at 37°C, the wells were washed three times with TBS-T, blocked with 5% BSA in TBS for 1 h at room temperature, and washed three more times with TBS-T. Various quantities of reduced PDI were then added in 100 μl volumes to each well for 1 h at 37°C with shaking. After three washes with TBS-T, a monoclonal anti-CTA1 antibody [34] was added in 100 μl volume at 1:100 dilution in 1% BSA-TBS for 1 h at 37°C. Following three more washes with TBS-T, an HRP–conjugated goat anti-mouse IgG secondary antibody (Jackson ImmunoResearch) was added in 100 μl volume at 1:1000 dilution in 1% BSA-TBS for 1 h at 37°C. The wells were then washed three times with TBS-T and incubated for 5 min at room temperature with 50 μl of TMB substrate (GE Healthcare, Pittsburgh, PA). This was followed by the addition of 50 μl stop solution (2 N H_2_SO_4_) and measurement of absorbance at 450 nm with a BioTek (Winooski, VT) Synergy plate reader.

### Isotope-edited FTIR spectroscopy

Samples for FTIR measurements (70 μg of uniformly ^13^C-labeled CTA1 and/or unlabeled PDI) were lyophilized in 10 mM sodium borate buffer (pH 7.0) containing 100 mM NaCl and resuspended in D_2_O containing 1 mM GSH and 10% glycerol. Uniformly ^13^C-labeled CTA1-His_6_ was produced as previously described [26]. Spectra were collected using a Jasco 4200 FTIR spectrometer (Easton, MD) with a spectral resolution of 0.964 cm^−1^ and a set resolution of 1 cm^−1^. Absorbance spectra were obtained using the transmittance of the protein sample and the buffer alone as a background reference. Uniformly ^13^C-labeled CTA1 was used to achieve spectral resolution of PDI and CTA1 due to a spectral downshift of the amide I band of the labeled protein by approximately 45 cm^−1^ from unlabeled PDI [26]. The amide I components of PDI were assigned to certain secondary structures as follows: α-helix (1660–1650 cm^−1^), irregular (i.e. unordered) structure (1649–1638 cm^−1^), β-sheet (1637–1620 cm^−1^). Amide I components between 1700 and 1660 cm^−1^ were assigned to various types of turns and are labeled as ‘other’ structures, while those between 1619 and 1600 cm^−1^ were assigned to side chains and were not considered in secondary structure evaluations. Deconvolution of protein secondary structures was performed using Grams/AI software (Thermo Scientific, San Francisco, CA) as previously described in detail [26].

## Results

### The unfoldase property of PDI is not an enzymatic activity

To evaluate the link between CTA1 protease sensitivity and CT disassembly, we first ran protease sensitivity assays with thermolysin as the protease. CTA1 was incubated alone or with PDI for 1 h at 25, 30, or 37°C before transfer to an ice bath. After 10 min on ice, the protease was added for 1 h at 4°C. This protocol ensured possible temperature-dependent proteolytic activities did not contribute to the processing of CTA1. Protease addition after toxin pre-incubation with PDI also ensured potential proteolytic activities against PDI did not disrupt PDI–CTA1 interactions. Toxin samples were resolved by SDS/PAGE and visualized by Coomassie stain.

**Figure 1:**
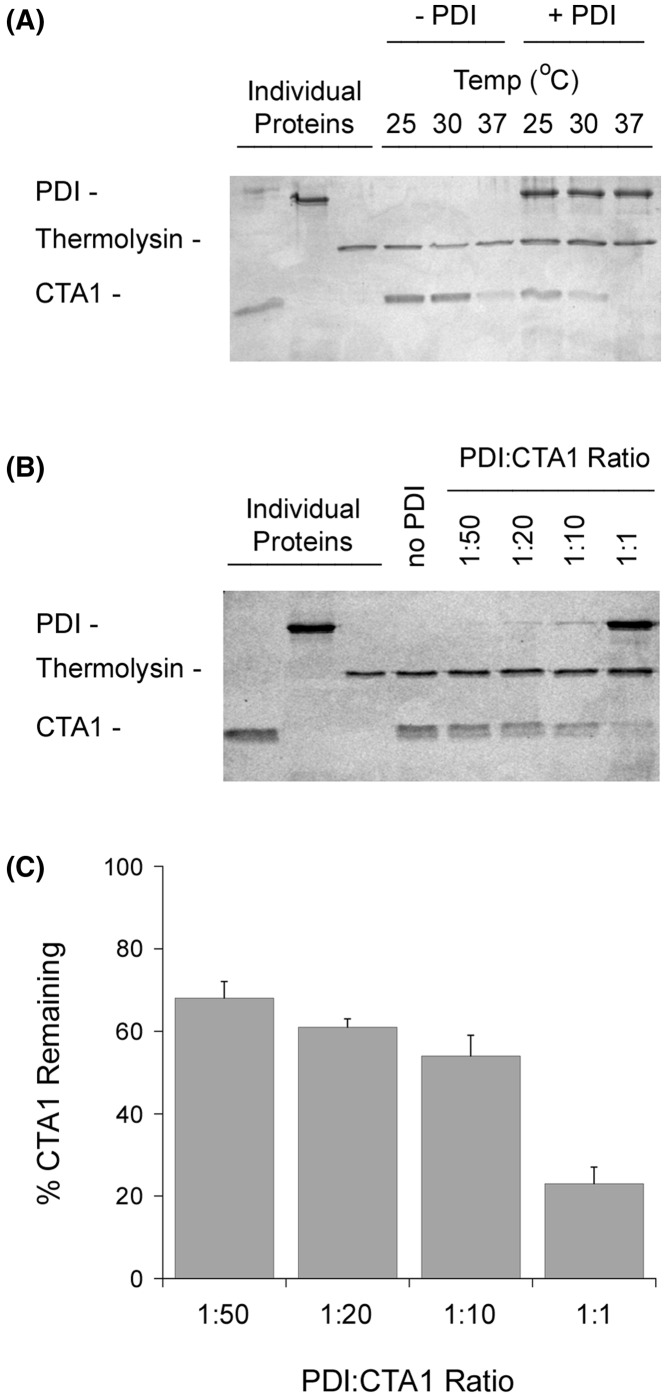
Effect of temperature and PDI on CTA1 protease sensitivity (**A**) CTA was placed in 20 mM sodium phosphate buffer (pH 7.4) with 1 mM GSH to reduce the CTA1/CTA2 disulfide bond. Toxin samples (1 μg) were then incubated at the indicated temperatures for 1 h in the absence or presence of equimolar PDI. All samples were subsequently placed on ice and exposed to thermolysin for 1 h at 4°C. Proteins were resolved by SDS/PAGE and visualized with Coomassie staining. Individual proteins (57 kDa PDI, 35 kDa thermolysin, and 21 kDa CTA1) representing the same quantity of material initially present in the experimental samples were also loaded, as indicated, in the far left lanes. (**B**) Samples of the reduced CTA1 polypeptide were incubated at 30°C for 1 h with different molar ratios of PDI. All samples were then placed on ice and exposed to thermolysin for 1 h at 4°C. Proteins were resolved by SDS/PAGE and visualized with Coomassie staining. Individual proteins representing the same quantity of material initially present in the experimental samples (1:1 ratio for PDI) were also loaded, as indicated, in the far left lanes. (**C**) Data from four replicate experiments represented by (B) were quantitated, with the thermolysin-treated toxin sample incubated in the absence of PDI set as the starting quantity of CTA1. Error bars report S.E.M.

The free CTA1 polypeptide is in a protease-resistant conformation at 25 and 30°C, but it switches to a protease-sensitive state when incubated at 37°C [27]. We confirmed this observation and further noted that CTA1 shifts to a protease-sensitive conformation at 25 or 30°C in the presence of PDI (Figure 1A). Proteolysis of the PDI-treated toxin was complete for the 37°C sample, although a minor amount of the 30°C sample and substantial amount of the 25°C sample remained after thermolysin treatment. These observations demonstrated CTA1 alone will assume a protease-sensitive state at 37°C, but it can also be converted into a protease-sensitive conformation at 25 or 30°C through its interaction with PDI.

Our initial protease sensitivity assay was performed with an equimolar ratio of PDI and CTA1. An enzymatic unfoldase activity should, however, allow low levels of PDI to act on CTA1. We addressed this issue by repeating our 30°C protease sensitivity assay with sub-stoichiometric levels of PDI. As shown in Figure 1B, low levels of PDI did not efficiently convert CTA1 into a protease-sensitive conformation. A 1:10 molar ratio of PDI:CTA1 left more than 50% of CTA1 in a protease-resistant state, while a 1:50 ratio left 70% of CTA1 in a protease-resistant state (Figure 1C). These results indicated the ‘unfoldase’ property of PDI is either a very poor enzymatic activity or is not an enzymatic function at all.

**Figure 2:**
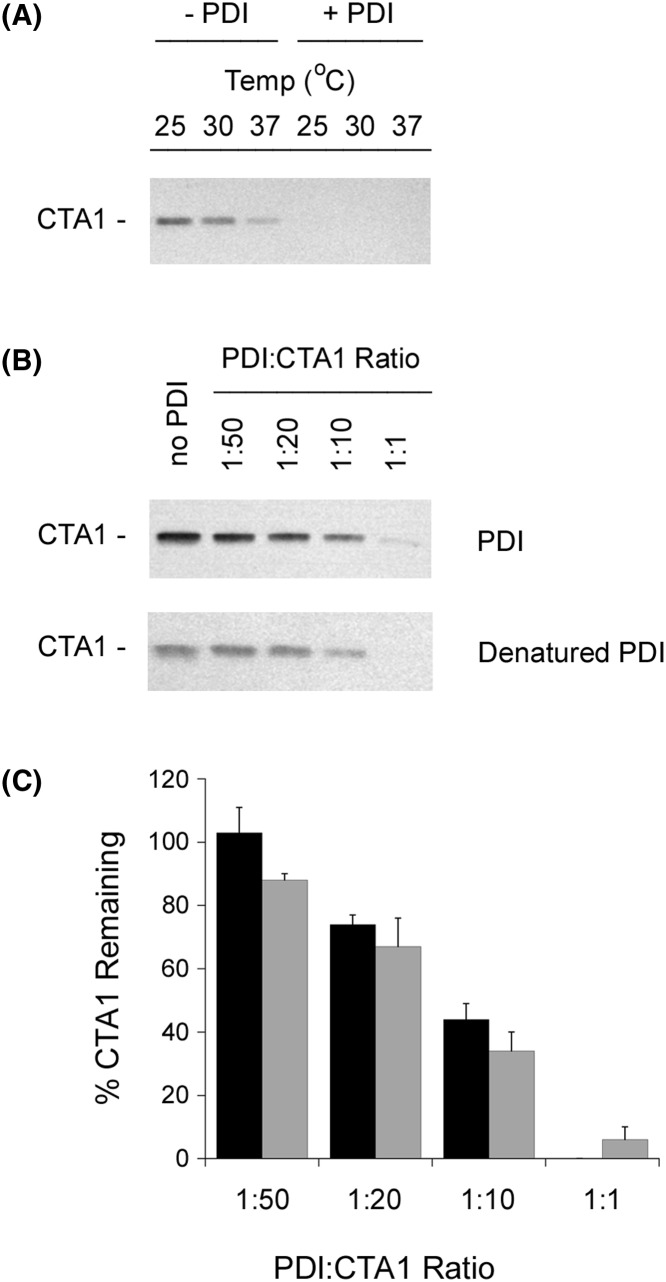
Effect of denatured PDI on CTA1 protease sensitivity (**A**) Samples of the reduced CTA1 polypeptide were incubated at the indicated temperatures for 1 h in the absence or presence of equimolar PDI. All samples were subsequently placed on ice and exposed to trypsin for 1 h at 4°C. SDS/PAGE with Western blot was used to detect CTA1. (**B**) Samples of the reduced CTA1 polypeptide were incubated at 30°C for 1 h with different molar ratios of PDI or heat-denatured PDI. All samples were then placed on ice and exposed to trypsin for 1 h at 4°C. SDS/PAGE with Western blot was used to detect CTA1. (**C**) Data from four replicate experiments represented by (B) were quantitated, with the trypsin-treated toxin sample incubated in the absence of PDI set as the starting quantity of CTA1. Black bars represent results obtained with denatured PDI, and gray bars represent results obtained with untreated PDI. Error bars report S.E.M.

Thermolysin recognizes hydrophobic amino acid residues [35], which makes it an appropriate protease to monitor protein folding. However, previous studies have used trypsin to probe the folding state of CTA1 [25,30,31]. We therefore repeated our protease sensitivity assay with trypsin as the protease. Since trypsin and CTA1 have similar molecular weights, these experiments used Western blot with a polyclonal CT antibody to follow the proteolysis of CTA1. We again found that CTA1 was in a protease-sensitive conformation when incubated alone at the physiological temperature of 37°C or when incubated with PDI at lower temperatures (Figure 2A). Efficient conversion of CTA1 into a trypsin-sensitive state did not occur with sub-stoichiometic levels of PDI (Figure 2B,C). The weak unfoldase activity of PDI at sub-stoichiometric molar ratios of PDI:CTA1 suggested the conversion of CTA1 into a protease-sensitive conformation resulted from a physical interaction with PDI rather than from an enzymatic function of PDI. To examine this possibility further, we tested the ability of denatured PDI to render CTA1 protease-sensitive. As shown in Supplementary Figure S3, the far-UV CD spectrum of PDI at 30°C displayed negative ellipticity in the 208–222 nm region that is indicative of a folded α-helix/β-sheet structure. Heating the protein to 90°C resulted in its unfolding, as evidenced by the loss of the 222-nm feature and a downshift of the 208-nm signal to 200 nm. The initial structure of PDI was not recovered after cooling to ambient temperature, implying an irreversible protein denaturation at 90°C. Yet, heat-treated PDI still converted CTA1 into a protease-sensitive conformation with similar efficiency to native PDI (Figure 2B,C). Denatured PDI could thus bind to CTA1, either from the retention of its CTA1-binding site or non-specific adherence. In either case, the unfoldase activity of PDI appears to be the by-product of a protein–protein interaction rather than an enzymatic function.

### The PDI-induced shift in CTA1 protease sensitivity does not contribute to CT disassembly

**Figure 3:**
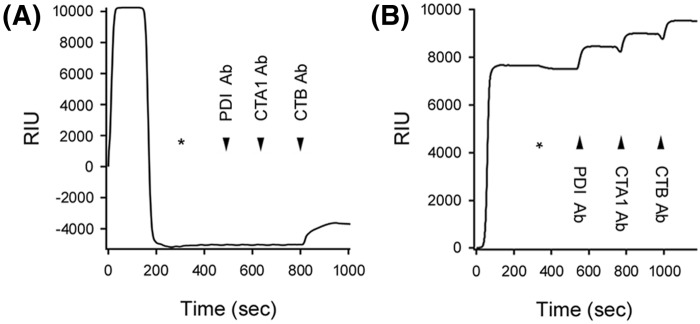
PDI-mediated holotoxin disassembly as monitored by SPR (**A**) PDI or (**B**) heat-denatured PDI was perfused over a CT-coated SPR sensor at 30°C in PBS-T buffer containing 1 mM GSH. PDI was removed from the perfusion buffer (denoted by the asterisks) and replaced with buffer containing sequential additions of anti-PDI, anti-CTA1, and anti-CTB antibodies as indicated by the arrowheads. Before initiating the experiment at time 0, a 10-min perfusion with PBS-T was used to generate a stable baseline corresponding to the mass of the sensor-bound CT holotoxin, which was set as zero RIU.

According to the unfoldase model, CT disassembly results from the PDI-mediated conversion of CTA1 into a protease-sensitive (i.e. putatively unfolded) conformation. Denatured PDI, which can still shift CTA1 to a protease-sensitive state, should therefore disassemble the reduced CT holotoxin. We tested this prediction with a real-time, SPR-based CT disassembly assay (Figure 3). After appending CT to a GM1-coated SPR sensor, a baseline measurement corresponding to the mass of the CT holotoxin was recorded and set to a zero RIU. PDI was then perfused over the sensor at 30°C under reducing conditions that cleaved the CTA1/CTA2 disulfide bond (1 mM GSH; Supplementary Figure S2). Addition of PDI to the SPR sensor generated an elevated RIU that indicated PDI had bound to the CT holotoxin. The subsequent PDI-mediated displacement of CTA1 from CTA2/CTB_5_ and resulting loss of both PDI and CTA1 from the SPR sensor were detected by a drop in RIU to a point below the initial baseline value. Sequential perfusions of anti-PDI, anti-CTA1, and anti-CTB antibodies over the sensor confirmed this interpretation, as only the anti-CTB antibody produced a positive signal (Figure 3A). Previous work using this system has shown: (i) reducing conditions alone do not result in the loss of CTA1 from the CT holotoxin; (ii) only reduced PDI will bind and displace CTA1 from CTA2/CTB_5_; and (iii) PDI only binds to CTA1 and not to CTA2/CTB_5_ [24,26]. Here, we found denatured PDI could bind to the CT holotoxin but could not dislodge reduced CTA1 from its non-covalent association with CTA2/CTB_5_: the elevated RIUs and positive signals obtained with anti-PDI, anti-CTA1, and anti-CTB antibodies at the end of this experiment indicated heat-denatured PDI remained associated with the intact CT holotoxin (Figure 3B). Thus, denatured PDI could induce CTA1 to assume a protease-sensitive state but could not facilitate disassembly of the CT holotoxin.

**Figure 4:**
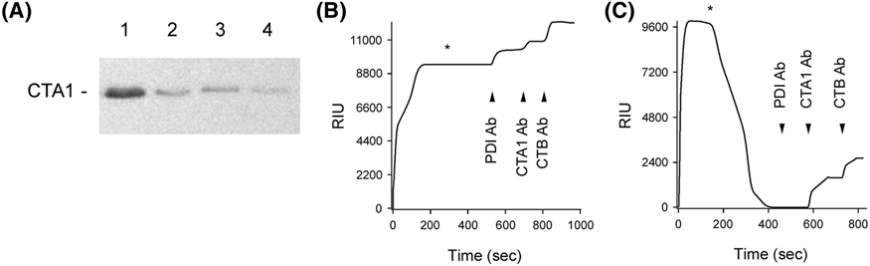
CTA1 protease sensitivity does not correspond to CT disassembly (**A**) Samples of the reduced CTA1 polypeptide were incubated at 30°C for 1 h in the absence of PDI (lane 1) or presence of equimolar PDI (lane 2), EDC-treated PDI (lane 3), or ribostamycin-treated PDI (lane 4). All samples were subsequently placed on ice and exposed to trypsin for 1 h at 4°C. SDS/PAGE with Western blot was used to detect CTA1. (**B**) EDC-treated PDI or (**C**) ribostamycin-treated PDI was perfused over a CT-coated SPR sensor at 30°C in PBS-T buffer containing 1 mM GSH. PDI was removed from the perfusion buffer (denoted by the asterisks) and replaced with buffer containing sequential additions of anti-PDI, anti-CTA1, and anti-CTB antibodies as indicated by the arrowheads.

Two other experimental conditions also documented the lack of correlation between PDI-induced protease sensitivity in CTA1 and PDI-mediated disassembly of the CT holotoxin (Figure 4). EDC-treated PDI and ribostamycin-treated PDI cannot displace CTA1 from its reduced holotoxin [24], so, according to the unfoldase model, neither drug-treated form of PDI should convert CTA1 into a protease-sensitive state. However, EDC-treated PDI and ribostamycin-treated PDI could both shift CTA1 to a trypsin-sensitive conformation at 30°C (Figure 4A). This indicated the conformational change in CTA1 does not result from the chaperone activity of PDI, as ribostamycin inhibits PDI chaperone function [13]. Along with the data recorded for denatured PDI, these observations suggested PDI binding alone is sufficient to convert CTA1 into a protease-sensitive state.

Because our previous CT disassembly assays with ribostamycin-treated PDI and EDC-treated PDI were performed at 37°C [24], we repeated those experiments here using the 30°C temperature of the protease sensitivity assay. EDC-treated PDI could stably associate with the reduced CT holotoxin but could not separate CTA1 from the rest of the toxin, as documented through the positive signals obtained with anti-PDI, anti-CTA1, and anti-CTB antibodies at the end of the experiment (Figure 4B). Ribostamycin-treated PDI could bind to CT but could not dislodge reduced CTA1 from its non-covalent association with CTA2/CTB_5_ (Figure 4C). The RIU signal returned to the baseline value corresponding to the mass of the CT holotoxin as soon as ribostamycin-treated PDI was removed from the perfusion buffer, and the antibody controls demonstrated CTA1 and CTB were present on the sensor whereas PDI was not. As also seen with denatured PDI (Figure 3B), these observations demonstrated there is no direct correlation between the conversion of CTA1 into a protease-sensitive conformation and CT disassembly.

### CT disassembly can still occur when CTA1 is in a protease-resistant conformation

**Figure 5:**
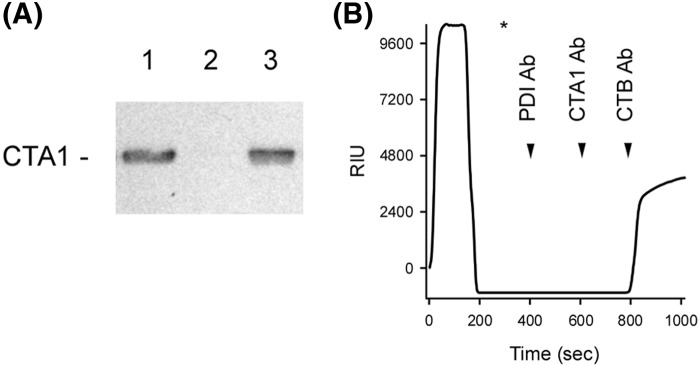
CTA1 protease resistance does not block CT disassembly (**A**) Samples of the reduced CTA1 polypeptide were incubated at 30°C for 1 h in the absence of PDI (lane 1), in the presence of equimolar PDI (lane 2), or in the presence of equimolar PDI treated with 10% glycerol (lane 3). All samples were subsequently placed on ice and exposed to trypsin for 1 h at 4°C. SDS/PAGE with Western blot was used to detect CTA1. (**B**) PDI in 10% glycerol was perfused over a CT-coated SPR sensor at 30°C in PBS-T buffer containing 1 mM GSH. PDI was removed from the perfusion buffer (denoted by the asterisks) and replaced with buffer containing sequential additions of anti-PDI, anti-CTA1, and anti-CTB antibodies as indicated by the arrowheads.

The unfoldase model also predicts CT disassembly will not occur when PDI cannot place CTA1 in a protease-sensitive conformation. The structure of CTA1 was stabilized with 10% glycerol [33] in order to test this prediction (Figure 5). Glycerol prevented PDI from converting CTA1 into a protease-sensitive conformation at 30°C (Figure 5A), although this treatment did not directly inhibit trypsin activity (Supplementary Figure S4). Glycerol thus affected the interaction between PDI and CTA1 rather than the proteolytic activity of trypsin. PDI could displace reduced CTA1 from the CT holotoxin in the presence of glycerol (Figure 5B), thus demonstrating CTA1 does not need to assume a protease-sensitive conformation in order for holotoxin disassembly to occur. Similar observations have been made for PDI–CT interactions at 4°C, a temperature that prevents PDI from converting CTA1 into a protease-sensitive conformation [30] but does not prevent the (albeit inefficient) disassembly of CT by PDI [26]. These collective results further emphasized the lack of correlation between the proposed unfoldase activity of PDI and the PDI-mediated separation of CTA1 from CTA2/CTB_5_.

### Glycerol does not inhibit the substrate-induced unfolding of PDI

**Figure 6:**
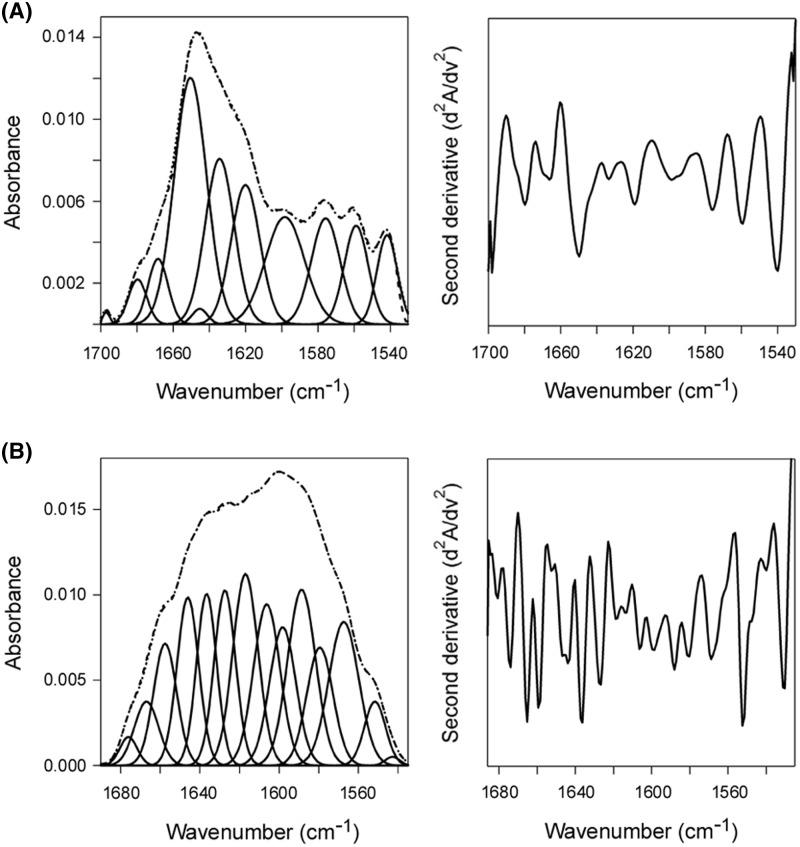
Glycerol does not inhibit the substrate-induced unfolding of PDI Curve fitting (left panels) and second derivatives (right panels) for the FTIR spectrum of reduced, glycerol-treated PDI recorded in the (**A**) absence or (**B**) presence of ^13^C-labeled CTA1 at 30°C. For curve fitting, the dotted line represents the sum of all deconvoluted components (solid lines) from the measured spectrum (dashed line). Deconvolution of the conformation-sensitive amide I bands determined that the secondary structure content of PDI alone contained 49 ± 2% α-helix, 36 ± 6% β-sheet, 6 ± 6% irregular, and 9 ± 1% other (mostly turn) structure. In the presence of CTA1, PDI contained 23 ± 2% α-helix, 37 ± 3% β-sheet, 26 ± 3% irregular, and 14 ± 3% other secondary structure. Values represent the averages ± S.D. of three to four separate curve fitting iterations.

Previous work using isotope-edited FTIR spectroscopy has shown that the secondary structure of CTA1 is not affected by its binding to PDI [26]. The unfolding of PDI itself serves as the foundation for our alternative ‘wedge’ model of CT disassembly. PDI unfolding should therefore occur in parallel with the separation of CTA1 from its holotoxin. Consistent with this model, we found that glycerol did not prevent the substrate-induced unfolding of PDI: isotope-edited FTIR spectroscopy demonstrated the secondary structure of glycerol-treated PDI contains 49% α-helix/36% β-sheet content in the absence of CTA1 (Figure 6A) and 23% α-helix/37% β-sheet content in the presence of CTA1 (Figure 6B). The loss of PDI α-helical structure resulting from its contact with CTA1 was accompanied by a corresponding 20% increase in PDI irregular (i.e. disordered) structure. This observation was remarkable, given the normal stabilizing effect of glycerol on protein structure. In contrast, we have shown EDC-treated PDI and ribostamycin-treated PDI do not unfold upon contact with CTA1 and do not disassemble the reduced CT [24] (Figure 4). These collective studies are consistent with the wedge model of CT disassembly in which the unfolding of PDI physically displaces reduced CTA1 from the CT holotoxin.

### A molar excess of PDI is required for conversion of holotxin-associated CTA1 into a protease-sensitive conformation and efficient disassembly of the CT holotoxin

**Figure 7:**
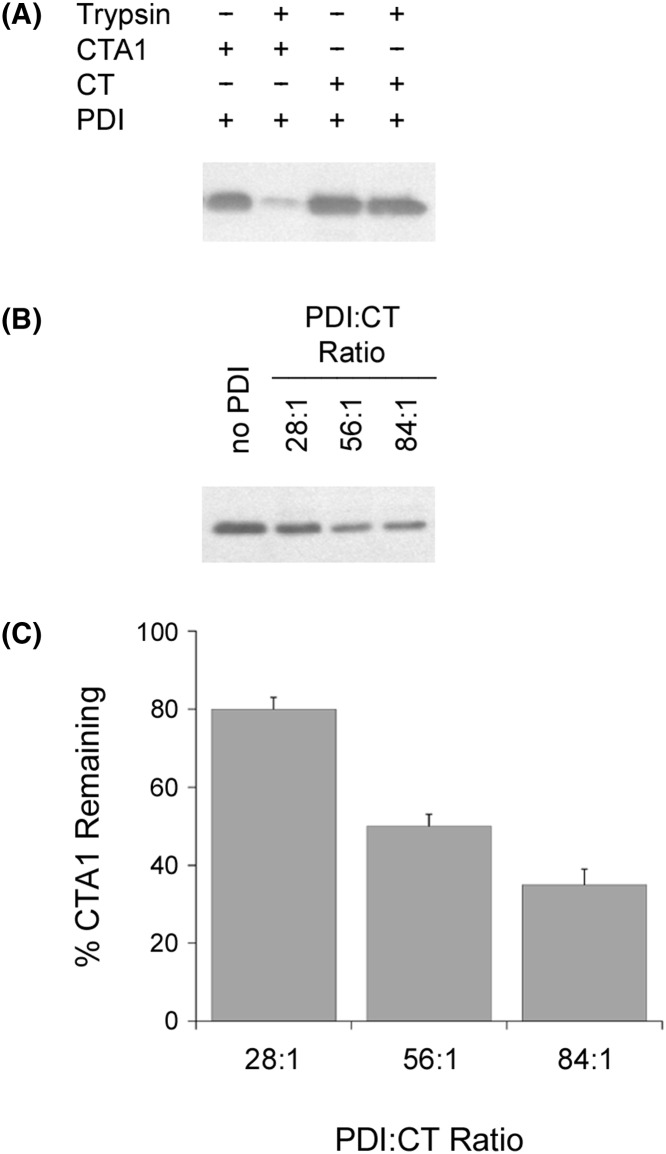
A molar excess of PDI is required to induce protease sensitivity in the holotoxin-associated CTA1 subunit (**A**) CT or CTA was placed in 20 mM sodium phosphate buffer (pH 7.4) with 1 mM GSH to reduce the CTA1/CTA2 disulfide bond. Toxin samples were incubated at 30°C for 1 h in the presence of equimolar PDI and were then shifted to 4°C for 1 h in the absence or presence of trypsin. CTA1 was resolved and visualized by SDS/PAGE with Western blot. (**B**) Samples of the reduced CT holotoxin were incubated at 30°C for 1 h with different molar ratios of PDI. All samples were then placed on ice and exposed to trypsin for 1 h at 4°C. CTA1 was resolved and visualized by SDS/PAGE with Western blot. (**C**) Data from four replicate experiments represented by (B) were quantitated, with the trypsin-treated toxin sample incubated in the absence of PDI set as the starting quantity of CTA1. Error bars report S.E.M.

Additional protease-sensitivity assays with the holotoxin-associated CTA1 subunit further emphasized the inefficient nature of the PDI unfoldase activity (Figure 7). Whereas a 1:1 molar ratio of PDI:CTA1 converted the free A1 subunit into a protease-sensitive conformation, holotoxin-associated CTA1 remained in a protease-resistant state after exposure to equimolar PDI (Figure 7A). Only 20% of holotoxin-associated CTA1 was converted into a protease-sensitive conformation by a 28-fold molar excess of PDI, and even an 84-fold excess of PDI was not sufficient for full conversion of holotoxin-associated CTA1 into a protease-sensitive conformation (Figure 7B,C). This may explain why the original study on the unfoldase activity of PDI used a 140-fold molar excess of PDI over CT holotoxin for the protease sensitivity assay [25].

**Figure 8:**
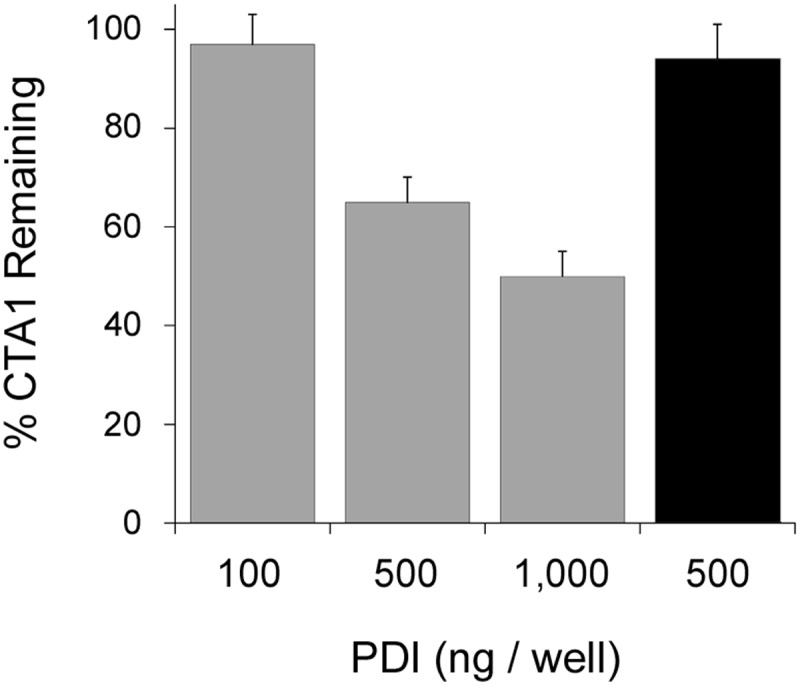
CT disassembly by PDI is an inefficient process The CT-coated wells of an ELISA plate were exposed to the stated quantities of reduced PDI (gray bars) or oxidized PDI (black bar) for 1 h at 37°C before extensive washing and sequential incubations with a monoclonal anti-CTA1 antibody and HRP–conjugated secondary antibody. All values were expressed as percentages of the signal recorded for untreated CT. Error bars report S.E.M. from four independent experiments.

The SPR platform can record CT disassembly in real-time, but it cannot determine the relative concentrations of CT and PDI in our assay because the amount of CT appended to the sensor surface is not quantitated. An alternative ELISA-based assay was therefore used to approximate the quantity of PDI needed for CT disassembly (Figure 8). With this assay, 5 ng/µl of CT in 100 µl volume was appended to the GM1-coated wells of a 96-well plate; 100, 500, or 1000 ng of reduced PDI was then added to the toxin for 1 h at 37°C before extensive washing. Loss of CTA1 from the plate was detected with an anti-CTA1 monoclonal antibody and compared with control toxin that was incubated in the absence of PDI. We documented a dose-dependent effect of PDI on CT disassembly. Approximately 50% of CTA1 was displaced from the holotoxin by 1000 ng PDI, whereas disassembly did not occur with 100 ng of PDI. Oxidized PDI could not dissociate CTA1 from the rest of the toxin, which was consistent with previous reports demonstrating oxidized PDI neither binds to CTA1 nor disassembles the CT holotoxin [25,26]. If CT coating of the ELISA well was 100% efficient, this would mean a sub-stoichiometric amount of PDI (0.28 PDI: 1.0 CT) could not dislodge CTA1 from its holotoxin while a 2.8-fold molar excess of PDI was only partially effective at holotoxin disassembly. These molar ratios represent a low-end estimate of the actual stoichiometry, as coating of the ELISA well was likely much less than 100% efficient. Our data thus indicate a molar excess of PDI over CT is required for effective holotoxin disassembly, which is consistent with previous reports that documented only weak CT disassembly with at least a minimum 90-fold [30] or 140-fold [25] molar excess of PDI over CT holotoxin. These collective observations again argue against an enzymatic mechanism for the displacement of reduced CTA1 from its holotoxin.

## Discussion

CTA1 must dissociate from CTA2/CTB_5_ to manifest its *in vivo* activity. Reduction in the A1/A2 disulfide bond is required for holotoxin disassembly, but reduced CTA1 does not spontaneously separate from CTA2/CTB_5_ [22,23]: PDI is responsible for displacing reduced CTA1 from its non-covalent assembly in the CT holotoxin [24–26]. This event represents a critical step in CT intoxication and could be related to the PDI-mediated disassembly of other AB toxins [36–40].

Contact with PDI shifts CTA1 from a protease-resistant conformation to a protease-sensitive conformation, which is thought to represent the PDI-mediated unfolding of CTA1. It has been further proposed that this unfoldase activity allows PDI to displace CTA1 from the rest of the toxin [25]. No method other than an indirect protease sensitivity assay has been used to demonstrate the unfoldase activity of PDI, and no molecular model has been proposed to explain the phenomenon. Our structure/function analysis of PDI–CTA1 interactions found that PDI does not alter the secondary structure content of CTA1 [26]. Instead, PDI itself unfolds upon contact with CTA1 [24]. We have accordingly proposed a new ‘wedge’ model for PDI-mediated toxin disassembly in which the expanded size of unfolded PDI pushes between CTA1 and CTA2/CTB_5_ to dislodge CTA1 from its non-covalent association with the rest of the toxin. Conditions that block the substrate-induced unfolding of PDI consequently block CT disassembly [24]. As the wedge mechanism involves a physical rather than enzymatic process, it does not occur efficiently at sub-stoichiometric molar ratios of PDI:CT (Figure 8). This, however, is not a barrier to *in vivo* translocation: the concentration of PDI in the ER is thought to approach the millimolar range [41] based on a total cellular concentration of 20 μM [42], but it only takes 1 nM of CT to produce a saturated cAMP response from intoxicated cells [43]. Furthermore, only a minor fraction (approximately 5%) of cell-associated CT reaches the ER [43–45]. CT will therefore encounter a molar excess of PDI in the ER.

CTA1 was thought to be a highly stable protein when the unfoldase model of PDI function was initially proposed [46–48], and protease sensitivity is usually indicative of an unfolded protein conformation. The original unfoldase model of PDI function was therefore reasonable at the time, but more recent studies have documented the intrinsic thermal instability of the free CTA1 subunit [27,33,49,50]. Thus, PDI is not required to unfold CTA1 at physiological temperature [27] and is not required for CTA1 to assume a protease-sensitive conformation at 37°C (Figures 1 and 2) [27,33]. Yet PDI does induce CTA1 to assume a protease-sensitive conformation at relatively low temperatures (25–30°C). This subtle conformational shift in tertiary structure could possibly account for the PDI-mediated release of CTA1 from CTA2/CTB_5_, but we have now discounted this possibility by documenting several instances in which there was no link between the apparent unfoldase activity of PDI (as monitored by CTA1 protease sensitivity) and the PDI-mediated separation of CTA1 from CTA2/CTB_5_ (which represents the only function assigned to the unfoldase activity of PDI). These collective observations indicate the conversion of CTA1 into a protease-sensitive state by PDI does not play a functional role in CT disassembly.

Our observations also demonstrate the unfoldase property of PDI is not an enzymatic function. Equimolar or greater ratios of PDI:substrate were required for both the shift in CTA1 protease sensitivity and CT disassembly. Furthermore, heat-denatured PDI could convert CTA1 into a protease-sensitive conformation with approximately equal efficiency to native PDI. We suggest results from the PDI-CTA1 protease sensitivity assay simply reflect the minor structural alterations to CTA1 tertiary structure that occur upon its binding to PDI rather than an actual unfolding event. Intermediate degradation products are not seen with the CTA1 protease sensitivity assay [25,31] (not shown), so this minor structural alteration may expose a single proteolytic site that, after proteolysis, reveals additional proteolytic sites and subsequently produces an all-or-nothing degradation event. Notably, a large number of potential cut sites for both thermolysin and trypsin are distributed throughout the CTA1 polypeptide (Supplementary Figure S5).

PDI was first proposed to exert an unfoldase effect on CTA1 in 2001 [25]. PDI interacts with several AB toxins and is involved with ER quality control [36–40,51], but no toxin or other protein has been shown to be unfolded by PDI since that initial report. With evolutionary concerns, it is impossible that PDI would only act as an unfoldase to facilitate the process of cholera intoxication. Our work here has shown the PDI-induced conversion of CTA1 into a protease-sensitive state is not an enzymatic process and is not functionally linked to CT disassembly. This leaves the unfoldase model without experimental support.

## Supporting information

**Figure S1.** The AB5 structure of cholera toxin A ribbon diagram derived from the crystal structure of CT (PDB 1S5F) is presented, with the 21 kDa A1 subunit in dark blue, the 5 kDa A2 subunit in light blue, and the 55 kDa B homopentamer in grey. The disulfide bond connecting CTA1 and CTA2 is highlighted in yellow.

**Figure S2.** Reduction of the CT holotoxin CT incubated in the absence (lane 1) or presence (lane 2) of 1 mM GSH for 5 min at 30°C was resolved by non-reducing SDS-PAGE and visualized with Coomassie stain. CT reduced with β- mercaptoethanol in the sample buffer was loaded as a reference in lane 3.

**Figure S3.** Heat denaturation of PDI Thermal unfolding of PDI was studied by CD spectroscopy using a 1.0 mm path-length quartz cuvette. The protein (800 nM) was dissolved in an aqueous pH 7.0 buffer containing 20 mM sodium borate, 100 mM NaCl, and 1 mM GSH. The initial spectrum was measured at 30°C (dark blue). The sample was then heated to 90°C, incubated for 5 min, and a spectrum was recorded at that temperature (red). The temperature was then decreased to 23°C, and a last spectrum was recorded (light blue). All spectra have been corrected by subtraction of the buffer spectrum.

**Figure S4.** Glycerol does not inhibit the enzymatic activity of trypsin Alpha-casein was placed in 20 mM sodium phosphate buffer (pH 7.4) containing 1 mM GSH. Protein samples (2 μg) were incubated for 1 h at 4°C in the absence or presence of trypsin before visualization with SDS-PAGE and Coomassie 713 stain. Samples exposed to trypsin were either untreated or co-incubated with 10% glycerol as indicated.

**Figure S5.** Location of cut sites for thermolysin and trypsin in CTA1 Recognition sites for thermolysin and trypsin along the length of the CTA1 polypeptide were identified with the ExPASy Peptide Cutter too.

## Abbreviations

CT: cholera toxin
EDC: 1-ethyl-3-(3-dimethylaminopropyl)carbodiimide
ER: endoplasmic reticulum
FTIR: Fourier transform IR
PBS-T: PBS (pH 7.4) with 0.05% Tween 20
PDI: protein disulfide isomerase
RIU: refractive index unit
SPR: surface plasmon resonance
TBS-T: TBS containing 0.1% Tween-20
