# Supplementary material for: Protein disulfide isomerase does not act as an unfoldase in the disassembly of cholera toxin
Source: Biosci Rep. 2018 Sep 7;38(5):BSR20181320. doi: 10.1042/BSR20181320 (PMC6127674; doi:10.1042/BSR20181320)
Supplement: Supplementary file 1 [file bsr20181320_Supp1.pdf]

SUPPLEMENTARY MATERIAL

Figure S1. The AB_5 structure of cholera toxin

A ribbon diagram derived from the crystal structure of CT (PDB 1S5F) is presented, with the 21 kDa A1 subunit in dark blue, the 5 kDa A2 subunit in light blue, and the 55 kDa B homopentamer in grey. The disulfide bond connecting CTA1 and CTA2 is highlighted in yellow.

Figure S2. Reduction of the CT holotoxin

CT incubated in the absence (lane 1) or presence (lane 2) of 1 mM GSH for 5 min at 30°C was resolved by non-reducing SDS-PAGE and visualized with Coomassie stain. CT reduced with β-mercaptoethanol in the sample buffer was loaded as a reference in lane 3.

Figure S3. Heat denaturation of PDI

Thermal unfolding of PDI was studied by CD spectroscopy using a 1.0 mm path-length quartz cuvette. The protein (800 nM) was dissolved in an aqueous pH 7.0 buffer containing 20 mM sodium borate, 100 mM NaCl, and 1 mM GSH. The initial spectrum was measured at 30°C (dark blue). The sample was then heated to 90°C, incubated for 5 min, and a spectrum was recorded at that temperature (red). The temperature was then decreased to 23°C, and a last spectrum was recorded (light blue). All spectra have been corrected by subtraction of the buffer spectrum.

Figure S4. Glycerol does not inhibit the enzymatic activity of trypsin

Alpha-casein was placed in 20 mM sodium phosphate buffer (pH 7.4) containing 1 mM GSH. Protein samples (2 μg) were incubated for 1 h at 4°C in the absence or presence of trypsin before

visualization with SDS-PAGE and Coomassie stain. Samples exposed to trypsin were either untreated or co-incubated with 10% glycerol as indicated.

Figure S5. Location of cut sites for thermolysin and trypsin in CTA1

Recognition sites for thermolysin and trypsin along the length of the CTA1 polypeptide were identified with the ExPASy Peptide Cutter tool.

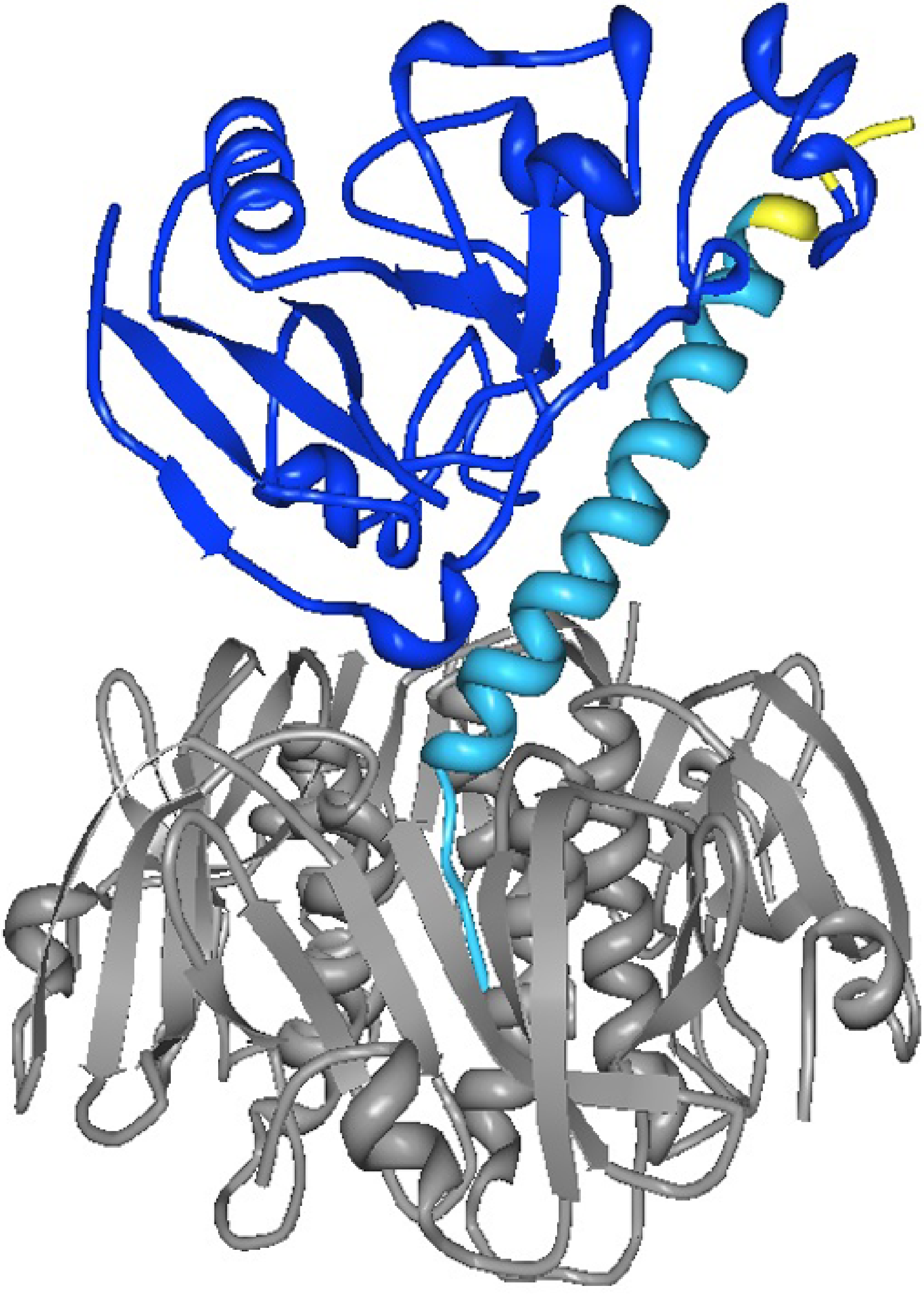

FIGURE S1

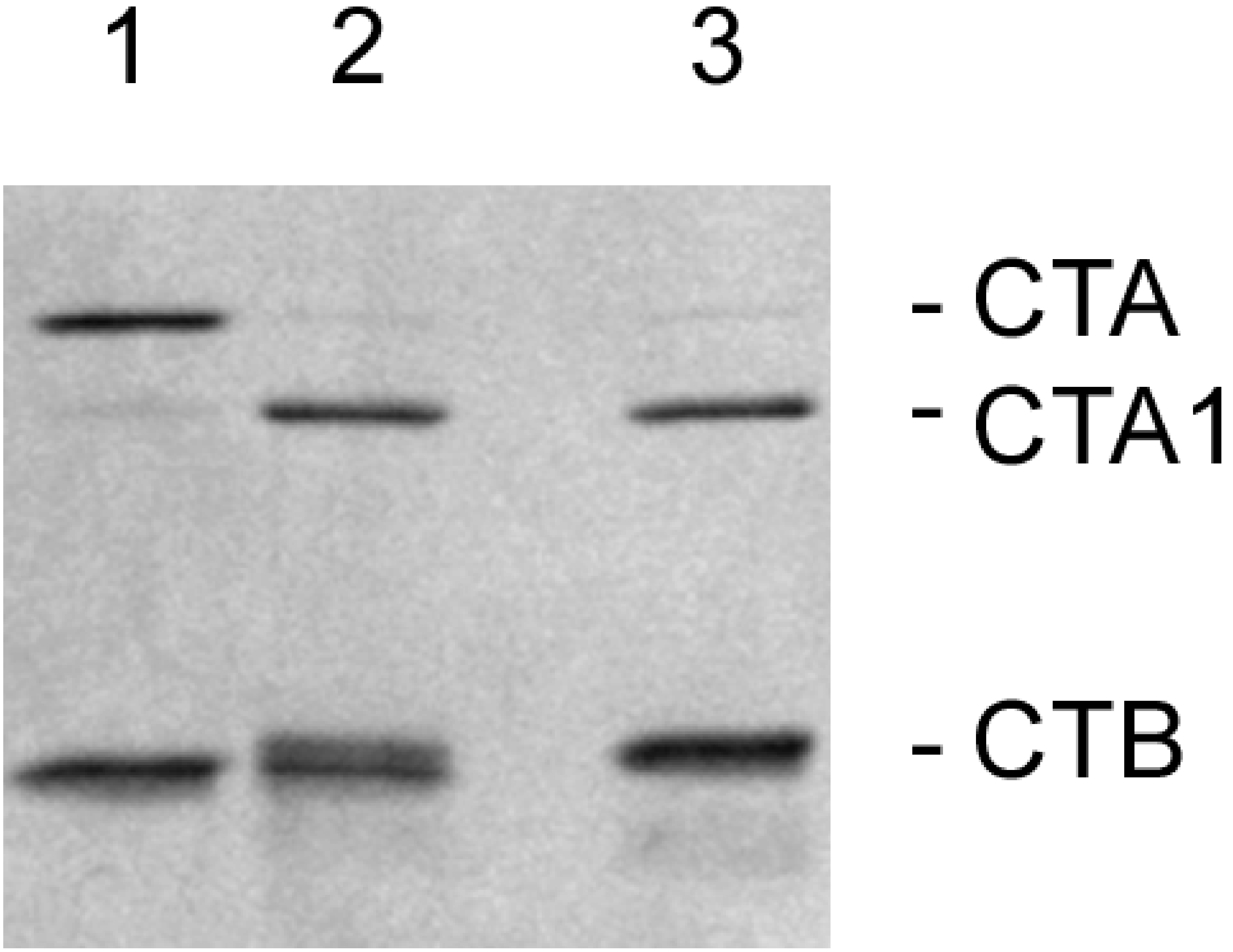

FIGURE S2

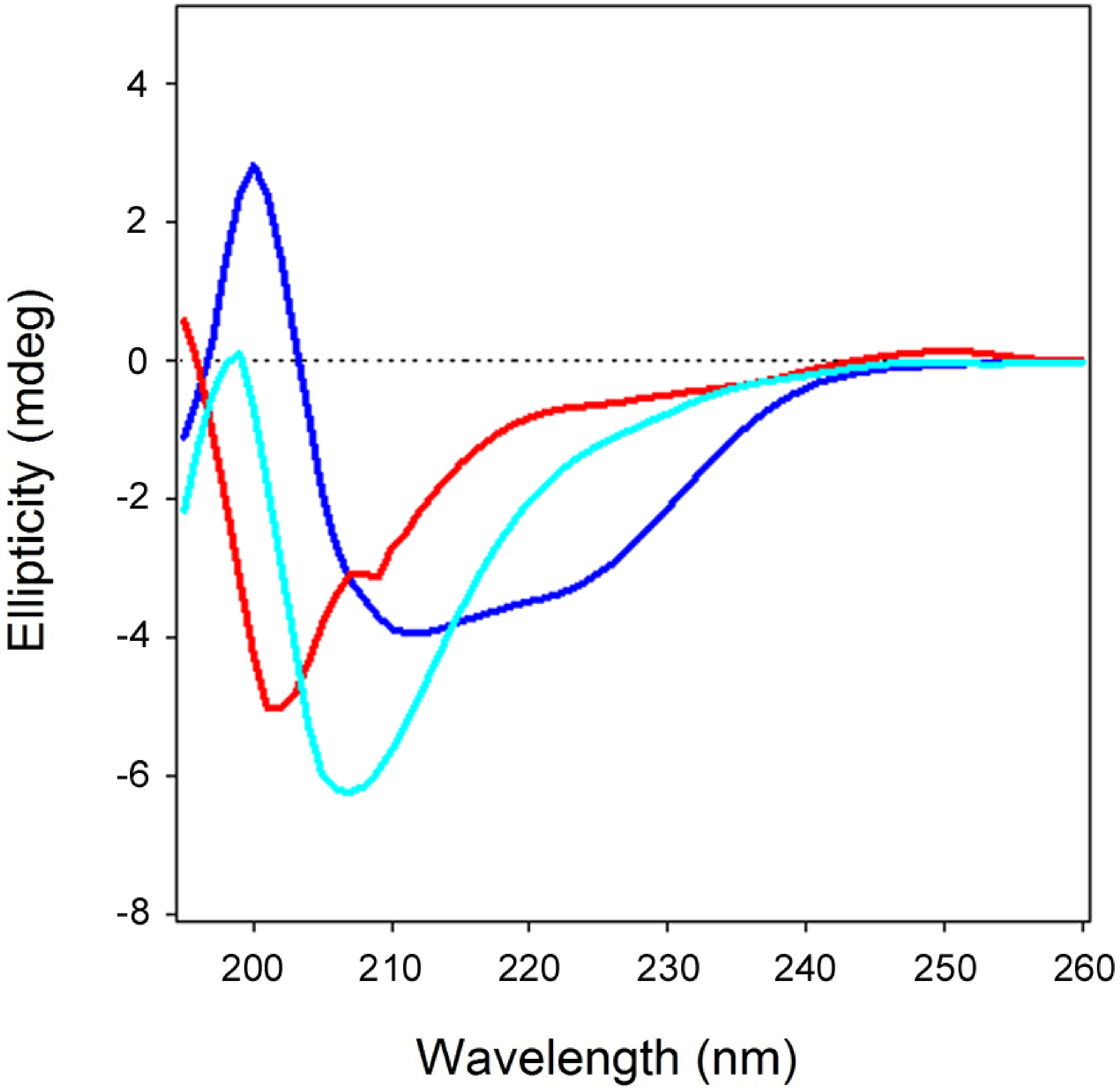
4
2
0
-2
-4
-6
-8
Ellipticity (mdeg)
200
210
220
230
240
250
260
Wavelength (nm)

FIGURE S3

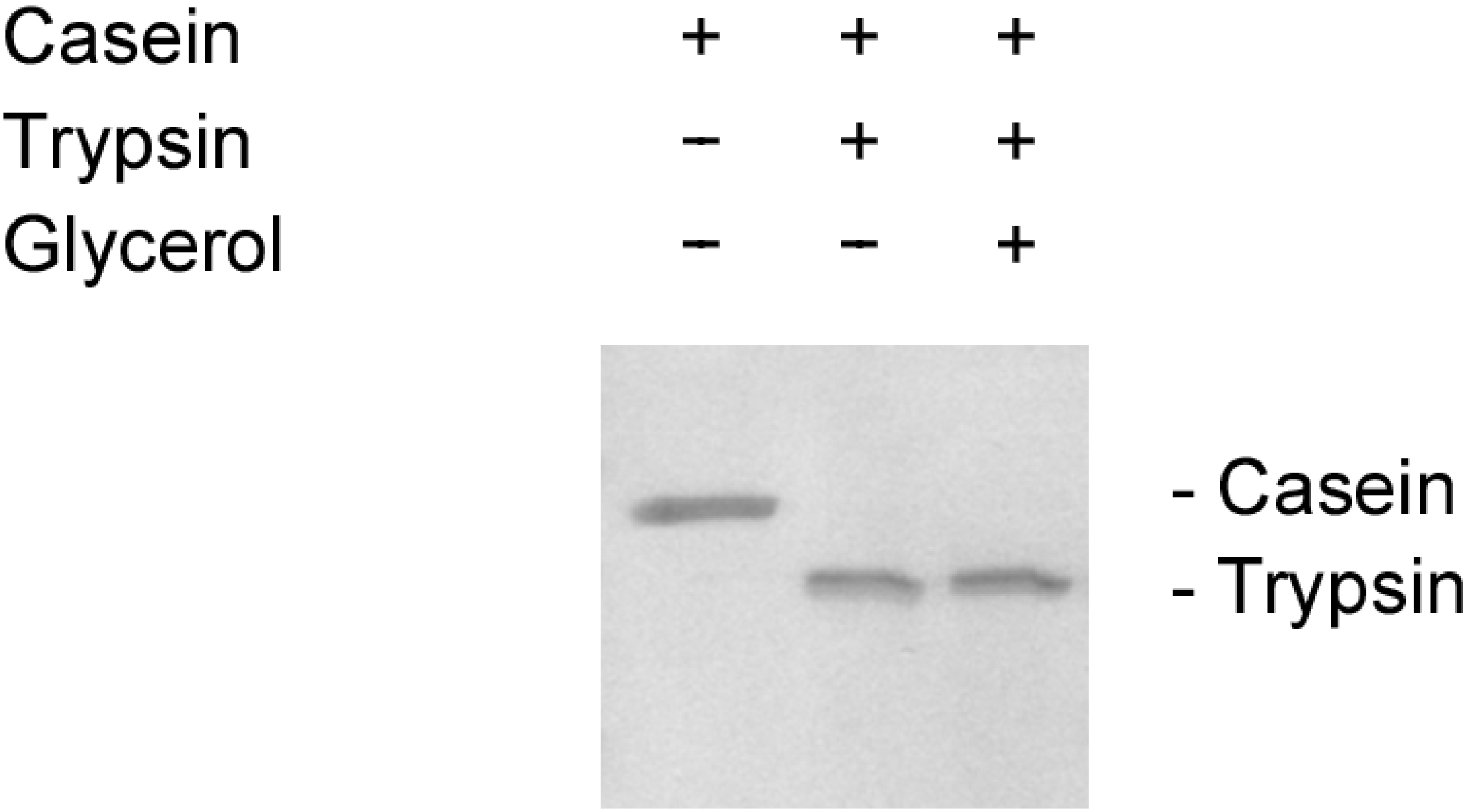

FIGURE S4

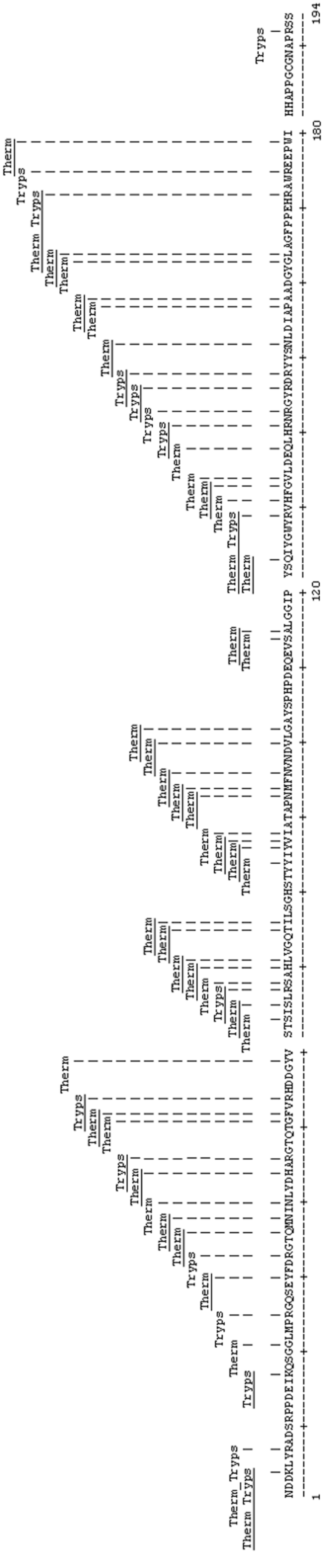

Therm
Tryps
NDDKLYRADSRPPDEIKQSGGLMPRGQSEYFDRGTQMNINLYDHARGTQTGFVRHDDGYV
STSISLRSAHLVGQTILSGHSTYYIYWIATAPNMFNVNDVLGAYSPHPDEQEVSALGGIP
YSQIYGWYRVHFGVLDEQLHRNRGYRDRYYSNLDIAPAADGYGLAGFPPEHRAWREEPWI
HHAPPGGNAPRSS
1
120
180
194

FIGURE S5
